# The Transcription Factor FgAtrR Regulates Asexual and Sexual Development, Virulence, and DON Production and Contributes to Intrinsic Resistance to Azole Fungicides in *Fusarium graminearum*

**DOI:** 10.3390/biology11020326

**Published:** 2022-02-18

**Authors:** Yanxiang Zhao, Huilin Sun, Jingwen Li, Chao Ju, Jinguang Huang

**Affiliations:** College of Plant Health and Medicine, Key Lab of Integrated Crop Disease and Pest Management of Shandong Province, Qingdao Agricultural University, Qingdao 266109, China; zhaoyx@qau.edu.cn (Y.Z.); 18053130618@163.com (H.S.); 18854807153@163.com (J.L.); juchao@qau.edu.cn (C.J.)

**Keywords:** plant pathogen, Fusarium head blight, mycotoxin, fungicide resistance, transcriptional regulation

## Abstract

**Simple Summary:**

*Fusarium graminearum* is a devastating plant pathogen that can cause wheat head blight. Azole fungicides are commonly used chemicals for control of this disease. However, *F. graminearum* strains resistant to these fungicides have emerged. To better understand the azole resistance mechanism of *F. graminearum*, we identified and characterized the Zn(II)2-Cys6 transcription factor FgAtrR in *F. graminearum.* We found that FgAtrR played critical roles in vegetative growth, conidia production, perithecium formation, and virulence on wheat heads and corn silks. FgAtrR was also involved in the resistance to azole antifungals by regulating the expression of the drug target FgCYP51s and efflux pump transporters. These results broadened our understanding of the azole resistance mechanisms of *F. graminearum*.

**Abstract:**

*Fusarium graminearum* is the predominant causal agent of cereal Fusarium head blight disease (FHB) worldwide. The application of chemical fungicides such as azole antifungals is still the primary method for FHB control. However, to date, our knowledge of transcriptional regulation in the azole resistance of *F. graminearum* is quite limited. In this study, we identified and functionally characterized a Zn(II)2-Cys6 transcription factor FgAtrR in *F. graminearum*. We constructed a FgAtrR deletion mutant and found that deletion of FgAtrR resulted in faster radial growth with serious pigmentation defects, significantly reduced conidial production, and an inability to form perithecia. The pathogenicity of the ΔFgAtrR mutant on wheat spikes and corn silks was severely impaired with reduced deoxynivalenol production, while the tolerance to prochloraz and propiconazole of the deletion mutant was also significantly decreased. RNA-seq indicated that many metabolic pathways were affected by the deletion of FgAtrR. Importantly, FgAtrR could regulate the expression of the FgCYP51A and ABC transporters, which are the main contributors to azole resistance. These results demonstrated that FgAtrR played essential roles in asexual and sexual development, DON production, and pathogenicity, and contributed to intrinsic resistance to azole fungicides in *F. graminearum*. This study will help us improve the understanding of the azole resistance mechanism in *F. graminearum*.

## 1. Introduction

*Fusarium graminearum*, a devastating fungal pathogen, causes Fusarium head blight in wheat and barley all over the world [1]. FHB results in significant grain yield loss, especially in recent years when climate and farming habits changed greatly [2,3]. Moreover, *F. graminearum* can produce mycotoxins, including deoxynivalenol (DON), nivalenol (NIV), and their acetyl derivatives (3-ADON, 15-ADON, and 4-ANIV), which pose serious threats to humans and animals [4,5]. Due to the lack of effective resistant cultivars, the application of chemical fungicides, including azole fungicides such as tebuconazole, is still the most commonly used and the most effective means of FHB control [6,7]. However, consecutive applications of azole chemicals have resulted in the emergence of *F. graminearum* strains resistant to these fungicides, which has been thrown into sharp focus [8].

The target of azole fungicide is the lanosterol 14α demethylase CYP51 (syn. ERG11), a cytochrome P450 protein [9,10]. CYP51 is a key enzyme in sterol biosynthesis, which is essential for the cell membrane, but azole drugs can block this process. Up to now, three azole resistance mechanisms have been extensively studied and reported: missense amino acid mutations of this target [11,12,13,14,15], overexpression of its coding genes resulting from an insertion in the promoter [16,17,18,19,20], and upregulation of ATP-binding cassette (ABC) superfamily and major facilitator superfamily (MFS) transporters [21,22,23,24,25]. Besides these, the roles of transcription factors in azole resistance have also received extensive attention [26,27,28,29,30]. Most of the transcription factors that regulate sterol biosynthesis in fungi are also involved in azole resistance. To date, two major regulatory mechanisms of fungal ergosterol biosynthesis have been proposed. One is mediated by the Zn(II)2-Cys6 transcription factor Upc2 and/or its homolog, found in *Saccharomyces cerevisiae* and *Candida* spp. [31,32,33]. For example, in the pathogenic yeast *Candida albicans*, Upc2 can regulate the expression of several sterol biosynthesis genes, such as *ERG2* and *ERG11*, when exposed to azole antifungals, while it has no effect on their basal transcription [32,34]. The other is dependent on the basic helix–loop–helix (bHLH) transcription factors known as sterol regulatory element-binding proteins (SREBPs), which were first identified in mammal cells, then in some fungi [27,35,36,37,38,39,40]. In the plant pathogenic fungus *Penicillium digitatum,* the SREBP-like transcription factor SreA is required for prochloraz resistance and is involved in the regulation of *CYP51* expression [27]. Similarly, SrbA in the human pathogenic fungus *Aspergillus fumigatus* also regulates the expression of *CYP51* and contributes to azole antifungal resistance [41]. In addition, transcription factors involved in the regulation of azole efflux pumps also conferred azole fungicide resistance. In *S. cerevisiae*, the zinc finger transcription factor Pdr1 and its paralog Pdr3 can bind to the promoter of the ABC transporter Pdr5 and are involved in pleiotropic drug resistance [42,43]. In *C. albicans*, the Zn(II)2-Cys6 transcription factor Tac1 regulates the expression of two azole efflux pumps belonging to the ABC transporters Cdr1 and Cdr2 [44,45]. Another zinc transcription factor, Mrr1, regulates the expression of the MFS transporter Mdr1 [46]. Both regulators are contributors to multiple drug resistance. Moreover, recently, several transcription factors functioning in both sterol biosynthesis and transporter expression were reported to respond to azole antifungals, such as AtrR, SltA, NctA, and NctB in *A. fumigatus*, and ADS-1 in *Neurospora crassa* [28,29,47,48]. Among these, AtrR is a Zn(II)2-Cys6 transcription factor with a GAL4-like Zn(II)2-Cys6 binuclear cluster DNA-binding domain and a fungal-specific transcription factor domain. AtrR can mediate resistance to azole drugs in *A. fumigatus* by binding to the AtrR response element (ATRE) in the *cyp51A* gene promoter to regulate the expression of the *cyp51A* gene, which has a similar function to the SREBP transcription factor SrbA, which is also a direct regulator of the *cyp51A* gene [29,41,49]. In addition, AtrR is also responsible for the regulation of the expression of the ABC transporter Cdr1B, an azole efflux pump.

In the phytopathogenic fungus *F. graminearum*, there are only a few research reports on transcription regulators related to azole resistance. Recently, Liu et al. found that sterol biosynthesis in *F. graminearum* may not be regulated by SREBP or Upc2 homologs, and they identified a novel Zn(II)2-Cys6 transcription factor, FgSR, involved in the transcriptional regulation of *FgCYP51A* and that contributed to azole resistance [30]. In addition to FgSR, the calcineurin-responsive transcription factor Fg01341 in *F. graminearum* was also reported to be associated with tebuconazole resistance [50]. To enrich our knowledge of the roles of transcription regulators in azole resistance, here, we identified and characterized the homolog of the Zn(II)2-Cys6 transcription factor AtrR in *F. graminearum*. These results revealed that FgAtrR played an essential role in asexual and sexual development, DON production, and pathogenicity. Importantly, FgAtrR could regulate the expression of the *FgCYP51A* gene and ABC transporter, and contributed to intrinsic resistance to azole fungicides in *F. graminearum*.

## 2. Materials and Methods

### 2.1. Strains and Culture Conditions

The wild-type strain PH-1 (NRRL 31084) of *F. graminearum* was used as the parental strain for this study. All *F. graminearum* strains were grown on potato dextrose agar (PDA), spezieller nährstoffarmer agar (SNA), or potato dextrose broth (PDB) liquid medium at 25 °C. To determine the production of conidia, these strains were cultured in carboxymethyl cellulose (CMC) liquid medium. All the strains were stored in 15% glycerol at −80 °C [51].

### 2.2. Construction of FgAtrR Deletion Mutants and Complemented Strains

The *FgAtrR* deletion mutants (ΔFgAtrR) were generated using the split-marker method [52]. First, the upstream and downstream fragments flanking the *FgAtrR* gene were amplified from genomic DNA of *F. graminearum* PH-1 with primer pairs FgAtrR-AF/AR and FgAtrR-BF/BR, respectively. Two segments of the hygromycin B phosphotransferase gene (*HPH*) were amplified from the pKH plasmid with primer pairs HYG-F/HY-R and YG-F/HYG-R. Then, the FgAtrR-flanking fragment and the *HPH* gene fragment were fused by overlapping PCR (Figure S1). The fused fragments were then introduced into the protoplast of *F. graminearum* PH-1 using a PEG-mediated protoplast transformation method [53]. Putative FgAtrR deletion mutants were identified from hygromycin-resistant transformants by PCR amplification with primer pairs FgAtrR-M-F/M-R, FgAtrR-K1-F/K1-R, and FgAtrR-K2-F/K2-R. The selected knockout transformants were further confirmed by Southern blot assay using the DIG High Prime DNA Labeling and Detection Starter Kit I (Roche Diagnostics, Mannheim, Germany) according to the manufacturer’s instructions.

For complementation, a plasmid was constructed by the yeast gap repair approach [54]. The fragment containing the full-length FgAtrR gene and its native promoter was amplified by PCR with the primer pair ComAtrR-F/R and then cotransformed with the *Xho*I-digested pYF11 vector into *Saccharomyces cerevisiae* strain XK1-25. The resulting recombinant plasmid was transformed into protoplasts of the ΔFgAtrR mutant using PEG-mediated fungal transformation, yielding the complemented strain ComFgAtrR.

For the analysis of the subcellular localization of FgAtrR, a similar method was used to generate strains expressing the FgAtrR-GFP fusion driven by the RP27 promoter. The difference was that the primer pair ComAtrR-GFP-F/R was used to amplify the fragment containing only the open reading frame of *FgAtrR* lacking a stop codon. All the primers used in this study are shown in Table S1.

### 2.3. Assessment of Conidial Production and Sexual Development

For evaluation of conidia production, five mycelium plugs (5 mm in diameter) of each strain were cultured for five days at 25 °C in CMC medium. The number of conidia was counted using a hemocytometer. For sexual reproduction assays, these strains were first cultured on carrot agar plates for 7 d, and then aerial hyphae were pressed down with 1 mL of sterile 2.5% Tween 60 solution as previously described [55]. Perithecium formation was examined after further incubation at 25 °C under black light for 2–3 weeks.

### 2.4. Pathogenicity Assays

In this assay, the FHB-susceptible wheat cultivar Xiaoyan22 was used to determine the pathogenicity of the *F. graminearum* strains PH-1, ΔFgAtrR, and ComFgAtrR to wheat. The wheat was grown in a phytotron at 50% relative humidity and at 25 °C in the day and 16 °C at night. This assay was performed as in a previous report [56] with minor modifications. Due to the low conidia production of FgAtrR deletion mutants, we could not harvest enough spores of ΔFgAtrR strains for experiments. Therefore, small mycelial plugs were used. Each strain was first grown on PDA plates for 3 days at 25 °C. At the wheat mid-flowering stage, small plugs of the same size taken from the edge of the colony of each strain was inoculated into a floret in the fifth or sixth spikelet from the base of a flowering wheat head. Ten flowering wheat heads were inoculated for each strain. The inoculated wheat head was kept moist for 48 h using a plastic bag. The infected spikelets of each wheat head were then recorded at 14 days postinoculation (dpi). The number of infected spikelets was used as the disease index to evaluate the virulence of different strains. In addition, the pathogenicity of the strain was also evaluated on corn silk of maize cultivar Zhengdan958. A 4-day-old mycelial plug of each strain was placed on one end of five fresh corn silks laying on wet sterile filter paper in a Petri dish and incubated at an incubator at 25 °C. The infection of corn silks was evaluated by the extent of discoloration after incubation for 7 days [57]. This experiment was repeated three times.

### 2.5. Analysis of DON Production

Each strain was first cultured in yeast extract–peptone–dextrose (YEPD) medium for 3 days, and then mycelia were collected. The mycelia (0.05 g) of each strain were broken into species with a grinder and then transferred to 50 mL trichothecene biosynthesis induction (TBI) medium [58]. After incubation at for 7 days, mycelia were collected by centrifugation, dried, and weighed. DON in the supernatant was determined by a competitive indirect ELISA method using a DON detection plate kit (Huanan Magtech Bio-Tech, Beijing, China), strictly following the manufacturer’s instructions. This experiment was repeated three times independently for each strain.

### 2.6. Azole Fungicide Sensitivity Testing

Radial growth was used to evaluate the sensitivity of *F. graminearum* strains PH-1, ΔFgAtrR, and ComFgAtrR to the 14α-demethylase inhibitors (DMIs) prochloraz and propiconazole as described previously [59]. A mycelial plug (5 mm in diameter) was taken from the margin of a 4-day-old colony and placed at the center of a PDA plate amended with tested DMIs dissolved in DMSO or DMSO alone of equal volume. The final concentrations of prochloraz were 0.02, 0.04, 0.08, 0.16, and 0.32 mg/l, and those of propiconazole were 0.1, 0.2, 0.4, 0.8, and 1.6 mg/l. Then, the plates were cultivated for 4 days. Colony diameter in each plate was measured in two perpendicular directions. For each plate, the true growth diameter of a colony was calculated using the averaged value subtracting the original mycelium plug diameter (5 mm). The resulting value was used to calculate the half-maximal effective concentration (observed EC50); namely, the fungicide concentration resulting in 50% mycelial growth inhibition. The experiment was repeated three times, and each strain was assayed in triplicate each time.

### 2.7. Microscopic Examinations

To localize FgAtrR, fresh hyphae that expressed the C-terminal GFP-tagged FgAtrR were examined using an OLYMPUS BX53 fluorescence microscope (OLYMPUS, Tokyo, Japan). For localization of nuclei, hyphae were first stained with 10 μg/mL 4’-6-diamidino-2-phenylindole (DAPI) (Solarbio, Beijing, China) for 10 min and then examined with a fluorescence microscope.

### 2.8. Quantitative Real-Time PCR (qRT-PCR) Assays

Total RNA was extracted from the mycelia of *F. graminearum* wild-type PH-1 and FgAtrR deletion mutants, using the SteadyPure Universal RNA Extraction Kit (Accurate Biology Co., Changsha, China). Ten micrograms of RNA were subjected to reverse transcription using HiScriptII Q RT SuperMix for qPCR (+gDNA wiper) (Vazyme Biotech, Nanjing, China). The expression level of each gene was determined by qRT-PCR using ChamQ SYBR qPCR Master Mix (Vazyme Biotech, Nanjing, China) with the primers listed in Table S1. For each sample, the *β-actin* gene (FGSG_07335) was used as the internal control. The relative expression level of the target gene was calculated using the 2^−^^ΔΔCq^ method [60]. This experiment was repeated three times independently, each with three replicates.

### 2.9. RNA Sequencing and Bioinformatics Analysis

Mycelia of *F. graminearum* wild-type strain PH-1 and ΔFgAtrR mutant were grown in PDB at 25 °C for 2 days and collected for RNA extraction (three biological replicates for each strain). RNA libraries were constructed using the NEBNext Ultra™ Directional RNA Library Prep Kit for Illumina following the manufacturer’s instructions, and sequenced on an Illumina HiSeq 6000 analyzer with the paired-end 2 × 150 bp model at Tianjin Novogene Bioinformatics Technology Co., Ltd. (Tianjin, China). The high-quality reads were mapped onto the reference genome of *F. graminearum* strain PH-1 (GCA_900044135.3) with the HISAT2 program [61]. The number of reads mapped to each gene was counted with featureCounts (v1.5.0-p3) [62]. The differentially expressed genes (DEGs) were identified with the R package DESeq2 (1.20.0). Genes with an adjusted *p*-value *p_adj_* < 0.05 and log2-fold fold change (Log2FC > 1) found by DESeq2 were assigned as differentially expressed. The Kyoto Encyclopedia of Genes and Genomes (KEGG) database was used to annotate protein pathways. Enrichment analysis of Gene Ontology (GO) terms and KEGG pathways of DEGs were performed with the R package clusterProfiler (3.4.4) [63,64,65]. In all analyses, a term with a corrected *p*-value < 0.05 was considered significant.

## 3. Results

### 3.1. Identification of the AtrR ortholog in F. graminearum

**Figure 1:**
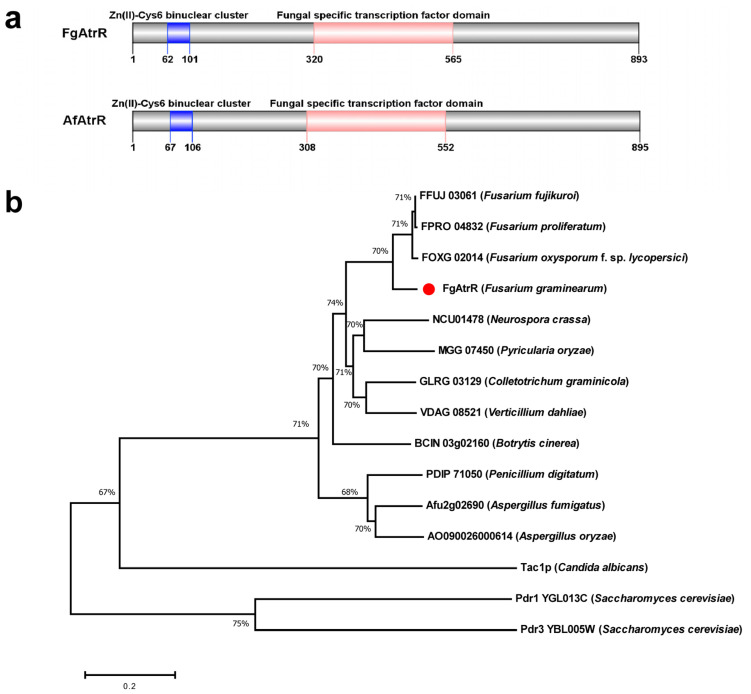
Domain structures and phylogenetic analysis. (**a**) Protein domains of FgAtrR and AfAtrR found in the Pfam database are shown. (**b**) The phylogenetic tree based on the amino acid sequence was constructed by the MEGA-X program using the neighboring-join method. The amino acid sequences from different species (shown in brackets) were aligned by MUSCLE.

A homology search of the genome of *F. graminearum* PH-1 by Ensemble BLASTp using the AtrR protein sequence of *A. fumigatus* (Afu2g02690) as the query revealed the highest sequence identity between locus FGSG_06810, here designated as FgAtrR. This gene encodes a protein of 893 amino acids containing an N-terminal Zn(II)2-Cys6 binuclear cluster domain followed by a fungal-specific transcription factor domain, which is similar to AfAtrR (Figure 1a) and shares 61% of sequence identity with AfAtrR. The homology search and multiple sequence alignment showed a high sequence identity with homologs in other filamentous ascomycetes, including *Fusarium* spp., *Neurospora crassa*, *Pyricularia oryzae*, *Botrytis cinerea*, and so on (Figure S2). However, few proteins with high sequence identity were found in other fungi, such as yeast, basidiomycetes, and zygomycetes. Phylogenetic analysis also suggested that AtrR is highly evolutionarily conserved in filamentous ascomycetes (Figure 1b).

### 3.2. FgAtrR Is Involved in Vegetative Growth and Pigmentation in F. graminearum

**Figure 2:**
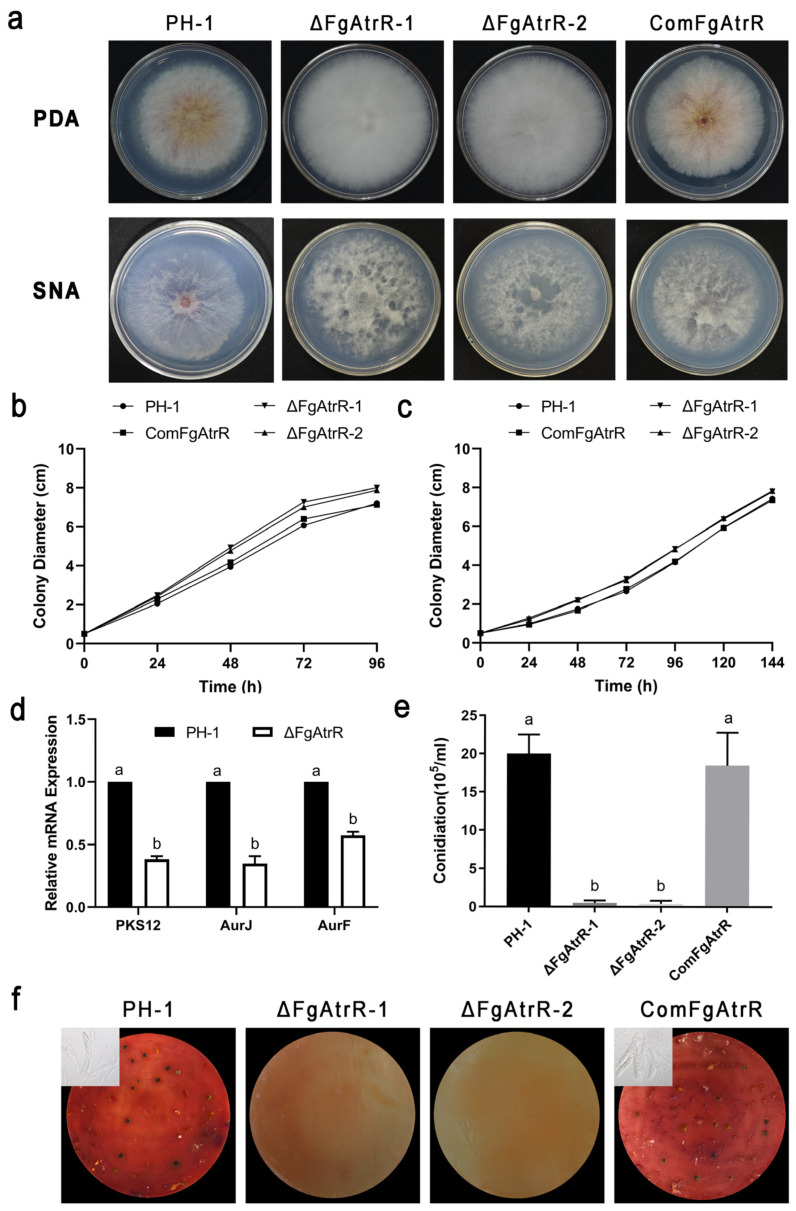
FgAtrR is involved in vegetative growth, pigmentation, and asexual and sexual reproduction in *F. graminearum*. (**a**) Colony morphology of the wild-type strain PH-1, the ΔFgAtrR mutants, and ComFgAtrR strain. Strains were grown on PDA plates at 25 °C for 4 days. (**b**) The radial growth rate on PDA plates of the wild-type strain PH-1, ΔFgAtrR, and ComFgAtrR. These strains were cultured on PDA plates for 4 days at 25 °C, and the colony diameters of each strain were measured every 24 h. (**c**) The radial growth rate on SNA plates of the wild-type strain PH-1, ΔFgAtrR, and ComFgAtrR. These strains were cultured on PDA plates for 6 days at 25 °C, and the colony diameters of each strain were measured every 24 h. (**d**) Relative expression levels of pigment biosynthesis-related genes *PKS12*, *AurJ*, and *AurF* in each strain. The total RNA of each strain was extracted from mycelia collected after 48 h of incubation at 25 °C in PDB broth. The actin gene was used as the internal control, and the expression level of each gene in PH-1 was arbitrarily set to 1. (**e**) Perithecia and ascospore formation. Strains were first cultured on carrot agar for 7 d and then mock-fertilized to induce sexual reproduction using Tween 60 solution. Perithecia formation was examined after incubation for an additional 3 weeks. (**f**) Conidia production of each strain. Conidia were harvested from the CMC cultures after incubation at 25 °C and 180 rpm for 4 days. All experiments were repeated three times with three replicates each time. Linear bars denote standard errors of three experiments. The different letters on the bars indicate a significant difference at the *p* < 0.05 level.

To determine the function of FgAtrR in *F. graminearum*, we constructed the *FgAtrR* deletion mutant (ΔFgAtrR) strain by a homologous recombination strategy. Two transformants, ΔFgAtrR-1 and ΔFgAtrR-2, which were confirmed by Southern blotting analysis (Figure S1), were used for further investigations. When the *F. graminearum* PH-1, ΔFgAtrR mutant, and ComFgAtrR were cultured on PDA agar plates for 96 h, the two mutants showed radial growth of 8.00 ± 0.07 cm and 7.88 ± 0.09 cm after 4 days at 25 °C. In contrast, wild-type and ComFgAtrR measured 7.21 ± 0.02 cm and 7.13 ± 0.08 cm, respectively. Namely, ΔFgAtrR grew approximately 10% faster than the wild-type PH-1 and ComFgAtrR strains (Figure 2a,b and Figure S3a). When these strains were grown on the low-nutrient SNA plates, a similar observation was obtained, indicating that FgAtrR might act as a negative regulator of the radial growth of *F. graminearum* (Figure 2a,c and Figure S3b). Moreover, the wild-type strain PH-1 and complemented strain ComFgAtrR could produce red pigments on PDA plates or in PDB medium, while ΔFgAtrR produced much less pigment and showed a white colony phenotype (Figure 2a and Figure S4). In order to explain this, a qRT-PCR assay was carried out to measure the expressions of key pigment-synthesis-related genes. The results indicated that the relative expression of *PKS12*, *AurJ*, and *AurF* in ΔFgAtrR was significantly decreased compared to wild-type PH-1 (Figure 2d).

### 3.3. FgAtrR Is Essential for Asexual and Sexual Development

The asexual and sexual reproduction of *F. graminearum* play important roles in the FHB disease cycle, so we wondered whether FgAtrR functioned in these development processes. To investigate the roles of FgAtrR in asexual development, conidial production by *F. graminearum* PH-1, ΔFgAtrR, and ComFgAtrR was evaluated in CMC medium. The results revealed that disruption of the *FgAtrR* gene could effectively block conidia production in 4-day-old CMC cultures. After 4 days of incubation, only 5.00 ± 3.16 × 10^4^ and 4.00 ± 3.10 × 10^4^ macroconidia/mL were obtained from ΔFgAtrR-1 and ΔFgAtrR-2 mutants, while 2.00 ± 0.23 × 10^6^ and 1.85 ± 0.38 × 10^6^ macroconidia/mL in PH-1 and ComFgAtrR strains could be harvested, respectively (Figure 2e). We also assessed the sexual reproduction of these strains on carrot agar plates. After incubation for 15 days, the wild-type PH-1 and complemented strain ComFgAtrR could produce normal perithecia, asci, and ascospores. However, ΔFgAtrR mutants failed to produce any perithecia (Figure 2f). These results implied that FgAtrR plays a vital role in the development of both asexual and sexual development.

### 3.4. FgAtrR Is Necessary for Full Virulence in F. graminearum

**Figure 3:**
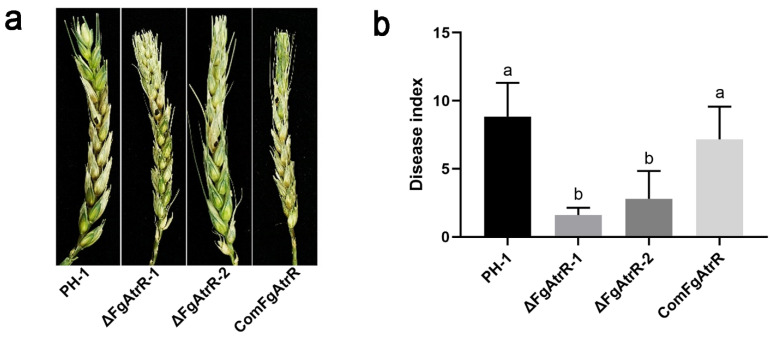
FgAtrR is required for full virulence of *Fusarium graminearum*. (**a**) The representative infected wheat heads were examined at 14 dpi with a 2 mm diameter mycelial plug of each strain. (**b**) The disease index was determined by the number of symptomatic spikelets per wheat head at 14 dpi. Ten flowering wheat heads were inoculated for each strain. Error bars showed the standard deviation. The same letters indicate no statistically significant difference at the *p* < 0.05 level.

Since *F. graminearum* is an important plant pathogen, we tested whether FgAtrR is involved in the pathogenicity of *F. graminearum*. The virulence assays were performed using flowering wheat heads and corn silks. It was found that the deletion mutant only infected 1–5 grains around the inoculation point, while the wild-type strain could infect 7–13 grains (Figure 3a,b). This indicated that the deletion of FgAtrR significantly impaired the virulence of *F. graminearum*. Similar results were also obtained in the corn silk assays. No obvious change in color was observed when silks were inoculated with the ΔFgAtrR strain compared to those inoculated with PH-1 strain or ComFgAtrR strain (Figure S5). The results above revealed that FgAtrR is crucial for full virulence in *F. graminearum*.

### 3.5. FgAtrR Is Required for DON Biosynthesis

**Figure 4:**
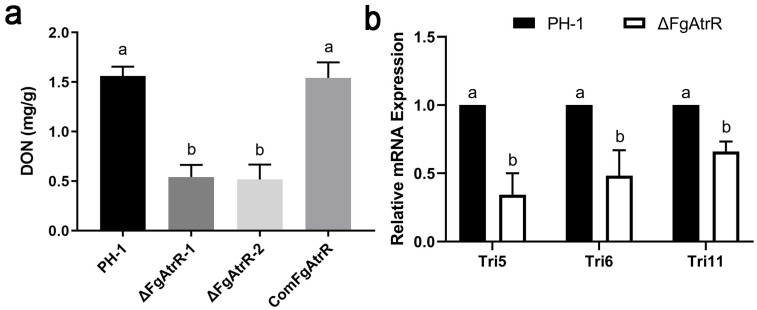
FgAtrR is vital for deoxynivalenol (DON) biosynthesis in *Fusarium graminearum*. (**a**) DON production level (per gram of dried mycelia) of each strain in TBI medium was measured. Error bars denote standard errors of three independent experiments. (**b**) Relative expression levels of DON biosynthesis-related genes *TRI5*, *TRI6*, and *TRI11* in each strain. The actin gene was used as the internal control, and the expression level of each gene in PH-1 was arbitrarily set to 1. The means of bars followed by different letters are significantly different at the *p* < 0.05 level.

DON is a critical virulence factor for the pathogenicity of *F. graminearum*. Therefore, we assessed the DON production of the *F. graminearum* PH-1, ΔFgAtrR, and ComFgAtrR in TBI medium. The wild-type strain PH-1 and complemented strain ComFgAtrR produced 1.56 ± 0.09 and 1.54 ± 0.16 mg/g mycelia after incubation for 7 days, respectively. In comparison, the two deletion mutants could only produce 0.54 ± 0.12 and 0.52 ± 0.15 mg/g of mycelia, which decreased by approximately 70% of DON production in the wild type (Figure 4a). The expression levels of several genes responsible for DON biosynthesis were also determined by qRT-PCR. The results showed that the transcription levels of *TRI5*, *TRI6*, and *TRI11* were significantly downregulated in the ΔFgAtrR mutant compared with those in the wild-type strain PH-1. These results suggested that FgAtrR modulates DON biosynthesis by regulating the expression of *TRI* genes in *F. graminearum* (Figure 4b).

### 3.6. FgAtrR Contributes to Intrinsic Resistance to Azole Fungicides

**Figure 5:**
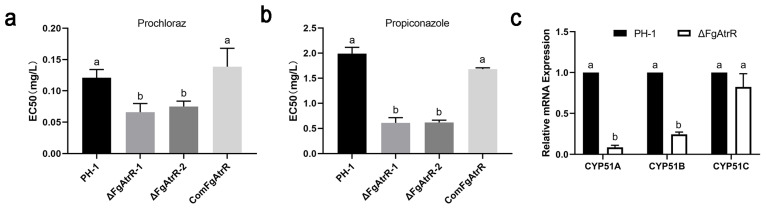
FgAtrR plays an important role in the response to azole fungicides of *Fusarium graminearum*. The EC50 value of prochloraz (**a**) and propiconazole (**b**) for *F. graminearum* PH-1, ΔFgAtrR, and ComFgAtrR strains were measured. Relative expression levels of azole fungicide target genes *CYP51A*, *CYP51B*, and *CYP51C* in each strain were also determined (**c**). The actin gene was used as the internal control, and the expression level of each gene in PH-1 was arbitrarily set to 1.

AtrR is an important regulator of azole resistance in *A. fumigatus* [49]. To investigate whether FgAtrR is involved in the response to azole chemicals in *F. graminearum*, we determined the sensitivity of the wild-type PH-1, ΔFgAtrR, and ComFgAtrR strain to the azole fungicides prochloraz and propiconazole. The EC50 value of prochloraz for wild-type PH-1 was 0.121 ± 0.013 mg/L, whereas it was 0.066 ± 0.014 mg/L and 0.075 ± 0.009 mg/L for each deletion mutant, which was only approximately 60% of that for the wild-type strain (Figure 5a and Figure S6a). Similar results were also obtained in the sensitivity test to propiconazole. The EC50 value of propiconazole for the deletion mutants (0.610 ± 0.104 mg/L and 0.617 ± 0.046 mg/L) was decreased to only approximately 30% of that for the wild type (1.990 ± 0.125 mg/L) (Figure 5b and Figure S6b). These results implied that deletion of FgAtrR significantly decreased the resistance of *F. graminearum* to prochloraz and propiconazole. The 14α-demethylase CYP51 is the main target of azole fungicide, so we further evaluated the transcription levels of three *CYP51* paralogous genes, *CYP51A*, *B*, and *C*, in *F. graminearum*. The results showed that compared to those in PH-1 strain, the expression of *CYP51A* was barely detectable, and *CYP51B* and *CYP51C* were downregulated by 66.2 ± 3.6% and 54.2 ± 2.7%, respectively, in ΔFgAtrR (Figure 5c), which suggested that FgAtrR has a critical role in the transcriptional regulation of *CYP51* genes in *F. graminearum*.

### 3.7. FgAtrR Mainly Localizes to the Nucleus

**Figure 6:**
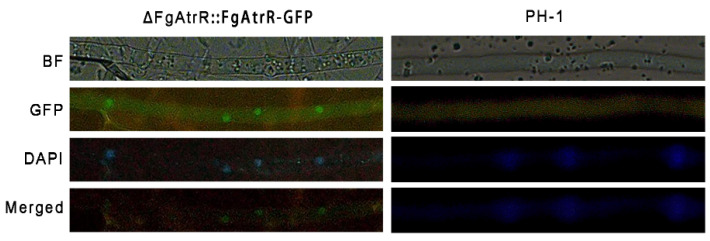
Subcellular localization of FgAtrR-GFP in *Fusarium graminearum*. Nuclei were stained with DAPI. The GFP and DAPI signals were examined under a fluorescence microscope. BF, bright field.

Sequence analysis suggested that FgAtrR harbors potential nuclear localization signal (NLS) sites, and may function as a transcription regulator. To determine the subcellular localization pattern of the FgAtrR protein in *F. graminearum*, we generated a strain that could express the FgAtrR-GFP fusion protein driven by the constitutive promoter RP27. Microscopic examination showed that the localization of green fluorescent signals could coincide with that of the well-known nuclear dye DAPI (4’-6-diamidino-2-phenylindole), indicating that FgAtrR-GFP predominantly localized to the nucleus (Figure 6).

### 3.8. RNA-seq Analysis with the ΔFgAtrR Mutant

**Figure 7:**
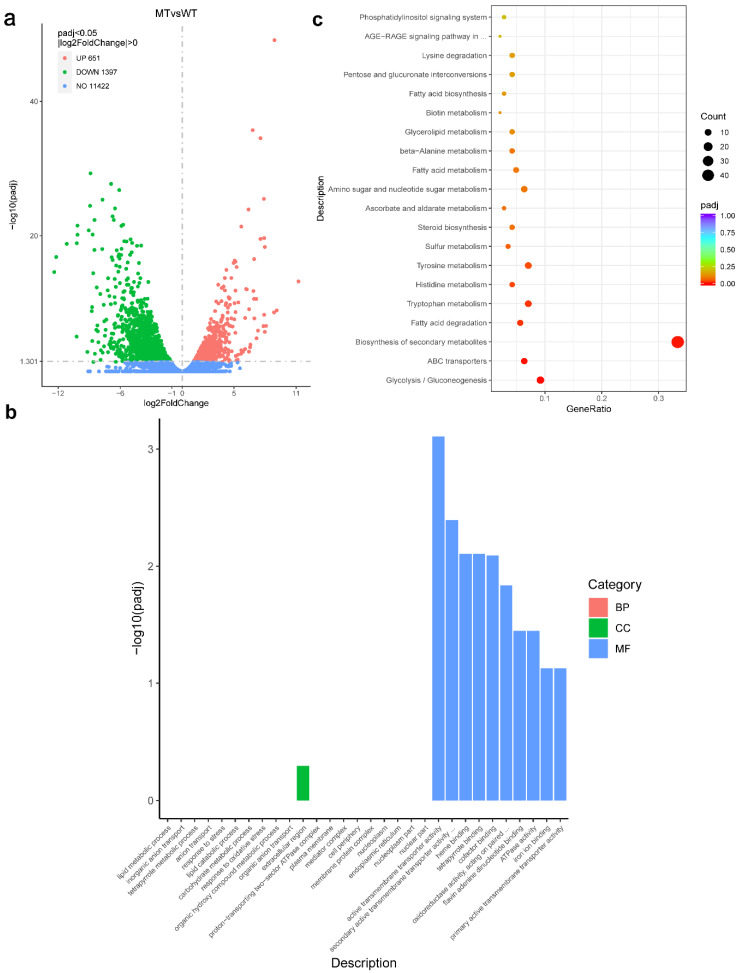
Transcriptome analysis of *Fusarium graminearum* wild-type strain PH-1 (WT) and FgAtrR deletion mutant strain (MT). (**a**) Volcano plot of identified transcripts. The dots in the upper left corner (labeled in green) are transcripts that were downregulated in the ΔFgAtrR strain, while the dots in the upper right corner (labeled in red) indicate those that were upregulated. (**b**) GO enrichment analysis of the downregulated genes in the ΔFgAtrR strain showed that 8 GO terms in molecular function (MF) were significantly enriched (*p_adj_* < 0.05). Detailed data are listed in Table S3. (**c**) KEGG enrichment analysis of the downregulated genes in the ΔFgAtrR strain showed that 7 KEGG pathways were significantly enriched (*p_adj_* < 0.05). Detailed data re listed in Table S4.

To identify genes regulated by FgAtrR, RNA-seq was carried out with RNA isolated from the *F. graminearum* PH-1 and ΔFgAtrR strains grown in PDB medium for 2 days. With the criteria of fold changes over 2, a total of 2048 differentially expressed genes (DEGs) were identified. Compared to the PH-1 strain, 1397 genes were upregulated, and 651 genes were downregulated in the ΔFgAtrR mutant strain (Figure 7a, Table S2). GO enrichment analysis of downregulated DEGs found that these downregulated genes were significantly enriched in eight molecular functions (MFs), and the most significant term was active transmembrane transporter activity (GO:0022804) (Figure 7b, Table S3). In KEGG enrichment, seven KEGG pathways were significantly enriched, including glycolysis/gluconeogenesis (fgr0010), ABC transporters (fgr02010), and biosynthesis of secondary metabolites (fgr01110) (Figure 7c, Table S4).

Among these DEGs, many genes were believed to be involved in growth and development or associated with the response to fungicide treatments in *F. graminearum* (Table 1 and Table S2). For example, a total of 6 DEGs were identified in the sterol synthesis pathway, which is essential for the fungal membrane structure, and all of them were downregulated. There were 16 ABC transporters identified as differentially expressed, all of which were downregulated as well, while six of them (FGSG_05076, FGSG_03882, FGSG_08309, FGSG_08312, FGSG_02316, and FGSG_08373) were reported to be differentially expressed after tebuconazole treatment [66]. Thirty-four genes whose products were annotated as major facilitator superfamily (MFS) proteins were differentially expressed. Among them, FGSG_00416 may affect the pathogenicity of *F. graminearum* [67]. Several secondary metabolite gene clusters were also affected by the deletion of *FgAtrR*, such as the C13/*PKS12* gene cluster and the putative C62 gene cluster [68]. All genes in the *PKS12* cluster (FGSG_02320~FGSG_02329), which is responsible for aurofusarin/rubrofusarin biosynthesis, were downregulated except for *AurR1* (FGSG_02320) and *AurS* (FGSG_02329) [69,70]. The C62 gene cluster (FGSG_02320~FGSG_02329) was considered to function during plant infection, and 6 of 11 genes in this cluster were downregulated [68].

## 4. Discussion

Azole fungicides are widely used in agricultural production. These drugs disturb ergosterol biosynthesis by inhibiting the activity of a cytochrome P450 enzyme, ergosterol 14α-demethylase (CYP51/ERG11). As mentioned above, the regulation of sterol metabolism plays significant roles in the resistance of fungi to azole chemicals. Many transcription factors involved in the regulation of sterol-biosynthesis-related genes and azole efflux pumps are associated with azole resistance. *F. graminearum* is a devastating plant pathogen. However, we have only superficial knowledge of transcriptional regulation during the azole response in this fungus. To date, only one transcription factor, FgSR, has been confirmed to directly modulate the expression of *FgCYP51A* by binding to the promoter region, thereby mediating the resistance of this fungi to tebuconazole [30]. In this study, we characterized the Zn(II)2-Cys6 transcription factor FgAtrR in *F. graminearum*, the homolog of the ABC transporter regulating the transcription factor AtrR in *A. fumigatus*. Microscopic examination confirmed the nuclear localization of FgAtrR-GFP in the hyphae of *F. graminearum*. Although the AtrR protein was first screened out using a DNA binding domain relating to *S. cerevisiae* Pdr1 and Pdr3 from *A. oryzae* [29], the phylogenetic analysis suggested that AtrR proteins are relatively distant from Pdr1 and Pdr3, but they are evolutionarily conserved in filamentous ascomycetes.

By comparing the wild-type strain PH-1 and ΔFgAtrR mutants, we found that deletion of FgAtrR led to faster radial growth, which suggested that FgAtrR is a potential repressor of radial mycelial growth. The white (but not red) colony color of the ΔFgAtrR mutant strain also implied that FgAtrR is involved in the regulation of aurofusarin biosynthesis, which is supported by the qPCR and RNA-seq data that 8 out of 10 genes involved in aurofusarin biosynthesis were significantly downregulated in the ΔFgAtrR mutants. Conidia and ascospores play pivotal roles in both the life cycle and disease cycle of *F. graminearum*. However, the ΔFgAtrR mutant produced only a few conidia and failed to produce perithecia and consequent ascospores, indicating that FgAtrR is essential for both asexual and sexual reproduction in *F. graminearum.* Taken together, FgAtrR plays an important role in the vegetative growth and reproduction of *F. graminearum*. Vdpf, a FgAtrR homolog in *Verticillium dahlia*, also functions in conidiation and the regulation of melanin biosynthesis-related genes, thereby influencing melanized microsclerotia formation [85]. This means that AtrR proteins in different fungi may have some conserved function.

*F. graminearum* is the primary pathogen of Fusarium head blight, and can also cause maize ear rot. In the virulence assays, we observed that the pathogenicity of ΔFgAtrR mutants on both wheat spikelets and corn silks was significantly decreased. Although the radial growth was enhanced, the infected spikelets were limited around the inoculation point on wheat heads inoculated with FgAtrR mutants. DON is a powerful toxin and pathogenic factor secreted by *F. graminearum* during plant infection, and has been shown to be crucial for the expansion of *F. graminearum* [86,87]. Compared to wild-type PH-1 and ComFgAtrR, the DON production of ΔFgAtrR mutants decreased significantly, which was probably due to the reduced expression of *TRI* genes. The product of *TRI5* is a vital enzyme that catalyzes the first biosynthetic step of trichothecene mycotoxins; TRI6 is a global transcription factor involved in trichothecene biosynthesis; and *TRI11*, encoding a C-15 hydroxylase, is also required in trichothecene biosynthesis [88]. However, the expression levels of all three genes were reduced significantly in ΔFgAtrR mutants in TBI medium, indicating that FgAtrR affected the synthesis of DON toxin by *F. graminearum*. Meanwhile, RNA-seq data revealed that many genes related to pathogenicity were differentially expressed between the PH-1 strain and the ΔFgAtrR mutant. For example, in our data, the chitinase FgChs8 was downregulated significantly in the deletion strain. FgChs8 has been reported to be essential for full virulence, and deletion of *FgCHS8* led to decreased pathogenicity with fewer infected spikelets on wheat heads [83]. Several cell-wall-degrading enzymes were also downregulated, such as Eng1, a putative endo-1,3(4)-b-glucanase Eng1 (FGSG_00184). The ΔEng1 deletion mutant exhibits restricted hyphal penetration and extension [89]. Six genes in the C62 gene cluster (FGSG_02320~FGSG_02329), which may also function in the plant infection stage, were downregulated as well [68]. Therefore, in addition to the decrease in DON production, the changes in the expression levels of these genes also contribute to the decrease in the virulence of the ΔFgAtrR mutant. In short, FgAtrR is necessary for the full virulence of *F. graminearum*.

Moreover, it is noteworthy that FgAtrR is involved in response to azole fungicides of *F. graminearum*. In *A. fumigatus*, the transcription factor AfAtrR regulates the expression of the azole target CYP51A and ABC transporter Cdr1B, and contributes to the resistance of *A. fumigatus* to azole antifungals [29]. Previous reports showed that the transcript levels of FgAtrR were significantly increased by tebuconazole treatment in *F. graminearum* [66]. We also found that the ΔFgAtrR mutant is more sensitive to the imidazole fungicide prochloraz. There are three CYP51 paralogs in *F. graminearum*. *FgCYP51B* is constitutively expressed and encodes the primary 14¦Á-demethylase. *FgCYP51A* also encodes a sterol 14α-demethylase and can be induced by azole treatment, while *FgCYP51C* is a *Fusarium*-specific gene and does not encode 14α-demethylase, but is required for full virulence [72]. All the expression levels of the three *CYP51* genes were significantly decreased in the deletion mutant compared with wild-type PH-1. In particular, we could hardly detect the expression of *FgCYP51A* in the ΔFgAtrR mutant, which was consistent with the RNA-seq data. This suggested that FgAtrR was involved in the azole resistance, probably by modulating the expression of *CYP51A* genes. Moreover, RNA-seq data showed that six genes involved in sterol biosynthesis; i.e., FGSG_11714, FGSG_05740, FGSG_03686 (*FgERG5B*), FGSG_04092 (*FgCYP51A*), FGSG_13888, and FGSG_06215, were all significantly reduced in the ΔFgAtrR mutant. In addition, the expression of FGSG_02814, a homolog of SREBP1, was greatly decreased in the ΔFgAtrR mutant, as was the expression of FGSG_00313, a homolog of Upc2. The function of these two genes needs to be further investigated. RNA-seq data also revealed that FgAtrR can regulate the expression of ABC transporters. *F. graminearum* contains 62 putative ABC transporter genes, 16 of which were downregulated in the ΔFgAtrR mutant, including FGSG_08312, which shares homology with Cdr1B in *A. fumigatus* and is directly regulated by AfAtrR. The decreased expression of the above genes may be an important reason for the difference in sensitivity of ΔFgAtrR to azole fungicides. These results suggested that FgAtrR conferred intrinsic azole resistance by coregulating sterol biosynthesis and the azole efflux pump.

In summary, the transcription factor FgAtrR exhibits various effects on *F. graminearum*, including diverse growth and development processes, DON toxin biosynthesis, pathogenicity, and resistance to azole fungicides. Although the direct interactions between FgAtrR and genes need to be further confirmed, FgAtrR can still be deemed an important transcription regulator for *F. graminearum* according to the currently known data. Characterization of the function of FgAtrR provided new clues regarding the resistance mechanism of *F. graminearum* to azole fungicides, and further study is needed to elucidate the detailed FgAtrR-mediated transcriptional regulation network.

## Figures and Tables

**Table 1:** List of several DEGs that may be involved in the phenotype mentioned above.

Protein Name	Gene Locus	Log2(Mutant/Wild-Type)	Reference
Sterol-biosynthesis-related genes			
FgERG6B	FGSG_05740	−5.52	[71]
FgERG5B	FGSG_03686	−4.87	[71]
FgCYP51A	FGSG_04092	−4.25	[72,73]
Aurofusarin biosynthesis gene cluster			
GIP3/AurO	FGSG_02321	−5.00	[69,74]
GIP4/AurT	FGSG_02322	−3.21	[69,74]
GIP5/AurR2	FGSG_02323	−3.03	[70]
PKS12	FGSG_02324	−5.88	[75,76]
GIP6/AurZ	FGSG_02325	−6.62	[74]
GIP7/AurJ	FGSG_02326	−5.62	[69,70]
GIP8/AurF	FGSG_02327	−5.88	[69,76]
GIP1	FGSG_02328	−5.05	[70,75]
ABC transporter			
ZRA1	FGSG_02139	−4.11	[77]
FgABC1	FGSG_10995	−3.15	[78]
	FGSG_08312	−2.16	[66]
	FGSG_05076	−3.63	[66]
	FGSG_02847	−2.49	[66]
	FGSG_08373	−1.89	[66]
Transcription factors			
	FGSG_10470	−3.41	[79]
	FGSG_00404	−1.36	[79]
Fpo1	FGSG_06651	1.26	[80]
MYT2	FGSG_07546	2.39	[81]
Others			
PDC1	FGSG_09834	−3.16	[82]
Chs8	FGSG_06550	−4.98	[83]
FgPKS7	FGSG_08795	−6.34	[84]
	FGSG_10608	−3.98	[68]
	FGSG_10609	−6.40	[68]
	FGSG_10611	−8.49	[68]
	FGSG_10612	−10.14	[68]
	FGSG_10613	−12.20	[68]
	FGSG_10614	−8.88	[68]

## Data Availability

RNA-seq read data were deposited in the NCBI Sequence Read Archive under accession no. PRJNA783513.

## Supplementary Materials

The following supporting information can be downloaded at: https://www.mdpi.com/article/10.3390/biology11020326/s1. Figure S1: Generation and identification of FgAtrR deletion mutants. Figure S2: Multiple sequence alignment of FgAtrR with homologs in other filamentous fungi. Figure S3: Deletion of FgAtrR promotes the radial growth of *Fusarium graminearum*. Figure S4: Deletion of FgAtrR blocks the aurofusarin biosynthesis of *Fusarium graminearum* in PDB medium. Figure S5: Deletion of FgAtrR impairs the pathogenicity of *Fusarium graminearum* on corn silks. Figure S6: The growth inhibition effect of azole fungicide prochloraz (a) or propiconazole (b) on the *Fusarium graminearum* PH-1, FgAtrR deletion mutants, and FgAtrR-complemented strain at different concentrations. Table S1: Primers used in this study. Table S2: All identified differentially expressed genes (DEGs). Table S3: GO enrichment analysis of downregulated DEGs. Table S4: KEGG enrichment analysis of downregulated DEGs.
