# Supplementary material for: The Transcription Factor FgAtrR Regulates Asexual and Sexual Development, Virulence, and DON Production and Contributes to Intrinsic Resistance to Azole Fungicides in Fusarium graminearum
Source: Biology (Basel). 2022 Feb 18;11(2):326. doi: 10.3390/biology11020326 (PMC8869466; doi:10.3390/biology11020326)
Supplement: Supplementary file 1 [file biology-11-00326-s001.zip › Supplementary Materials/Supplementary Figures.pdf]

A transcription factor FgAtrR regulates the asexual and sexual development, virulence, DON production and contributes to the intrinsic resistance to azole fungicides in *Fusarium graminearum*

Yanxiang Zhao[1†], Hunlin Sun[1†], Jingwen Li[1], Chao Ju[1], Jinguang Huang[1*]

[1] College of Plant Health and Medicine and Key Lab of Integrated Crop Disease and Pest Management of Shandong Province, Qingdao Agricultural University, Qingdao 266109, Shandong Province, China

[†] These authors contributed equally to this work.

Correspondence: jghuang@qau.edu.cn

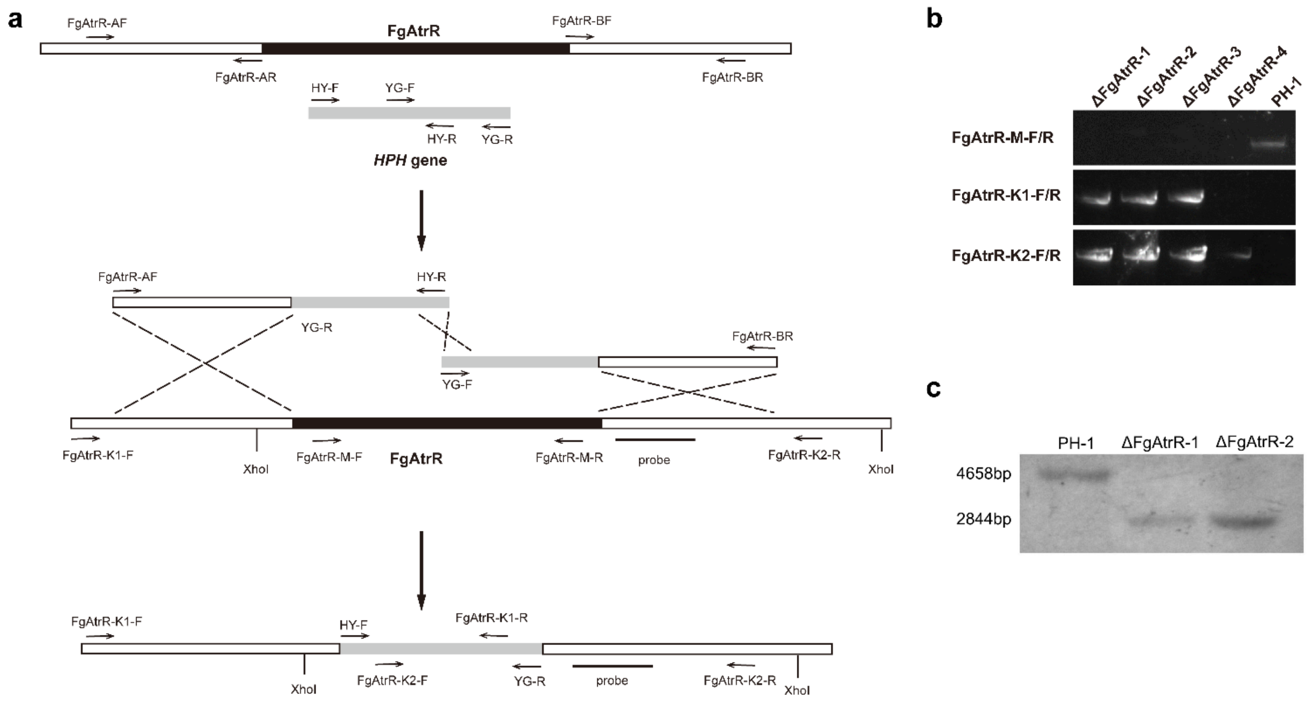

Figure S1 Generation and identification of FgAtrR deletion mutants. (a) Schematic representation of the gene deletion strategy. The *FgAtrR* gene was replaced by the hygromycin gene (*HPH*) through homologous recombination. Primer binding sites are labeled by arrows. (b) PCR analysis of transformants showed ΔFgAtrR-1, -2, and -3 might be the correct FgAtrR deletion mutants. (c) Southern blot analysis of FgAtrR deletion mutants and the wild-type strain PH-1 confirmed the ΔFgAtrR-1 and ΔFgAtrR-2 were corrected. A 524 bp downstream fragment was used as a probe. The genomic DNA sample of each strain was digested with *Xho*I.

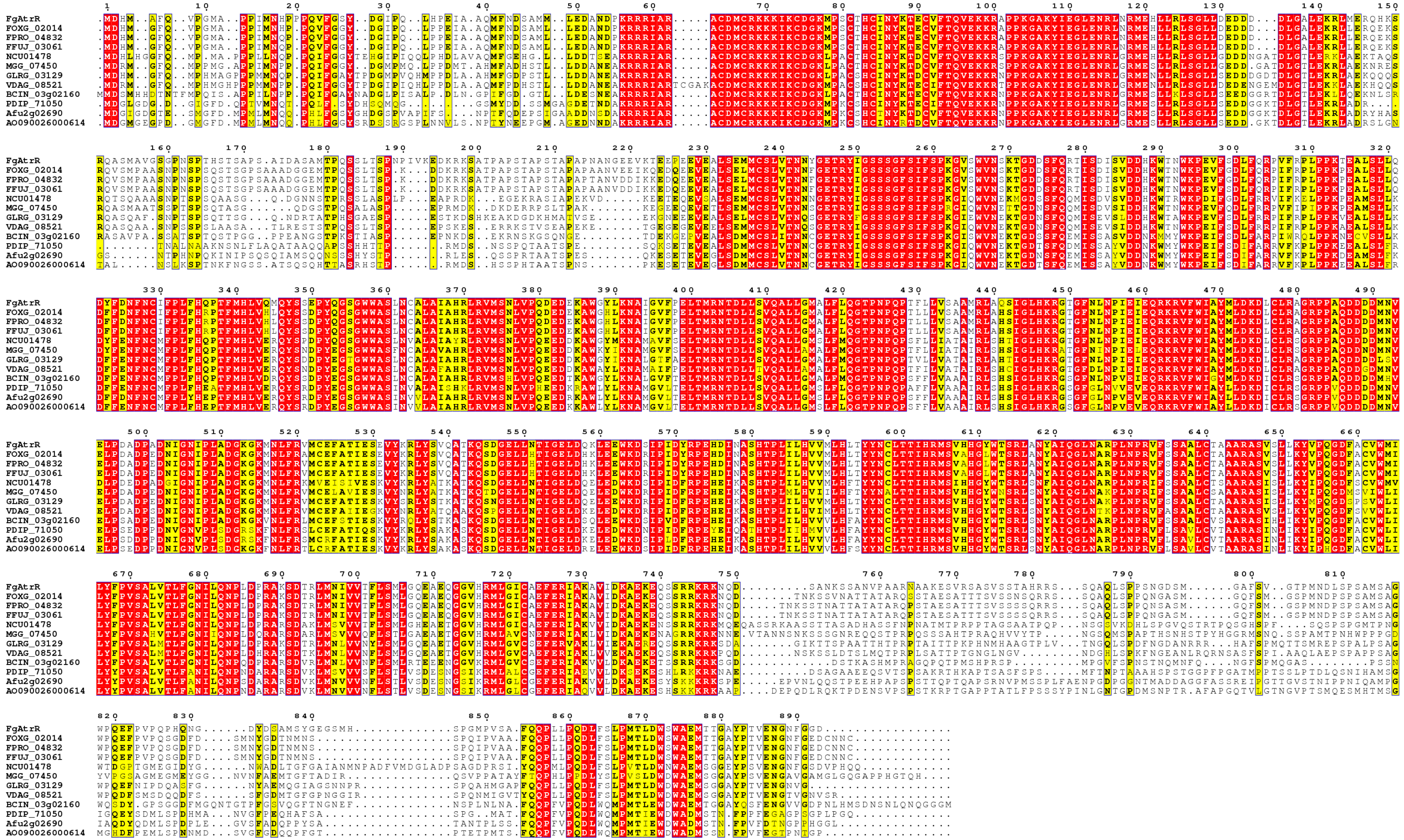
FgAtrR
FOXG_02014
FPRO_04832
FFUJ_03061
NCU01478
MGG_07450
GLRG_03129
VDAG_08521
BCIN_03g02160
PDIP_71050
Afu2g02690
AO090026000614

Figure S2 Multiple sequence alignment of FgAtrR with homologs in other filamentous fungi. Sequences for analysis are as follows: FgAtrR from *Fusarium graminearum*, FOXG_02014 from *F. oxysporum* f. sp. *lycopersici*, FFUJ_03061 from *F. fujikuroi*, FPRO_04832 from *F. proliferatum*, NCU01478 from *Neurospora crassa*, MGG_07450 from *Pyricularia oryzae*, BCIN_03g02160 from *Botrytis cinerea*, PDIP_71050 from *Penicillium digitatum*, Afu2g02690 from *Aspergillus fumigatus*, AO090026000614 from *A. oryzae*, VDAG_08521 from *Verticillium dahliae*, GLRG_03129 from *Colletotrichum graminicola*.

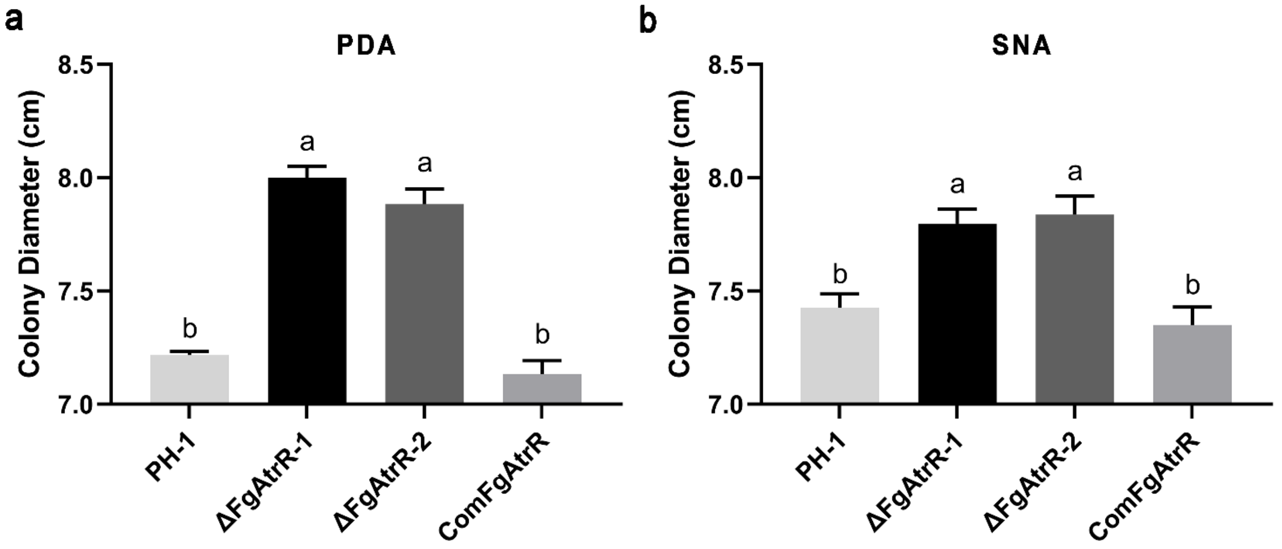

Figure S3 Deletion of FgAtrR promotes the radial growth of *Fusarium graminearum*. The colony diameter of each strain was measured after incubation for 96 h on PDA plates or 144 h on SNA plates. The experiment was repeated three times with three replicates each time. Linear bars denote standard errors of three experiments. The different letters on the bars indicate a significant difference at the $p < 0.05$ level.

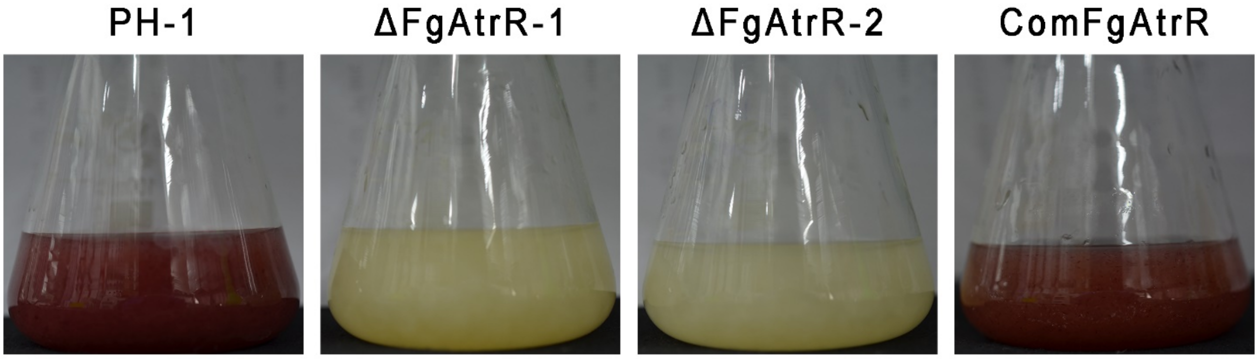

Figure S4 Deletion of FgAtrR blocks the aurofusarin biosynthesis of *Fusarium graminearum* in PDB medium. Each strain was cultured in flasks containing PDB medium at 25°C for 5 d.

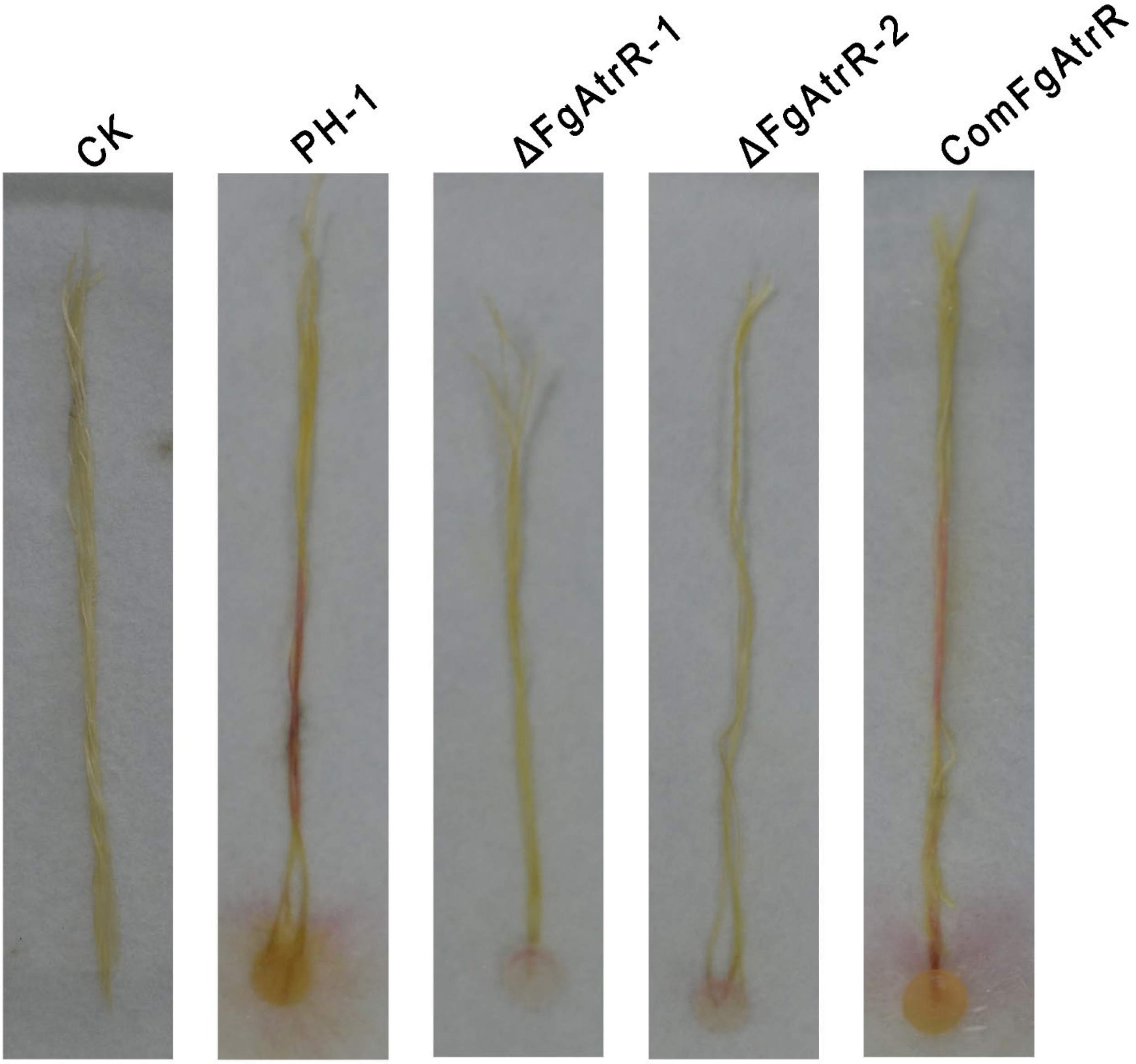

Figure S5 Deletion of FgAtrR impairs the pathogenicity of *Fusarium graminearum* on corn silks. A mycelial plug was placed at one end of a bundle of silk corn. The photographs were taken after 7 days of incubation in a humid chamber at 25 °C.

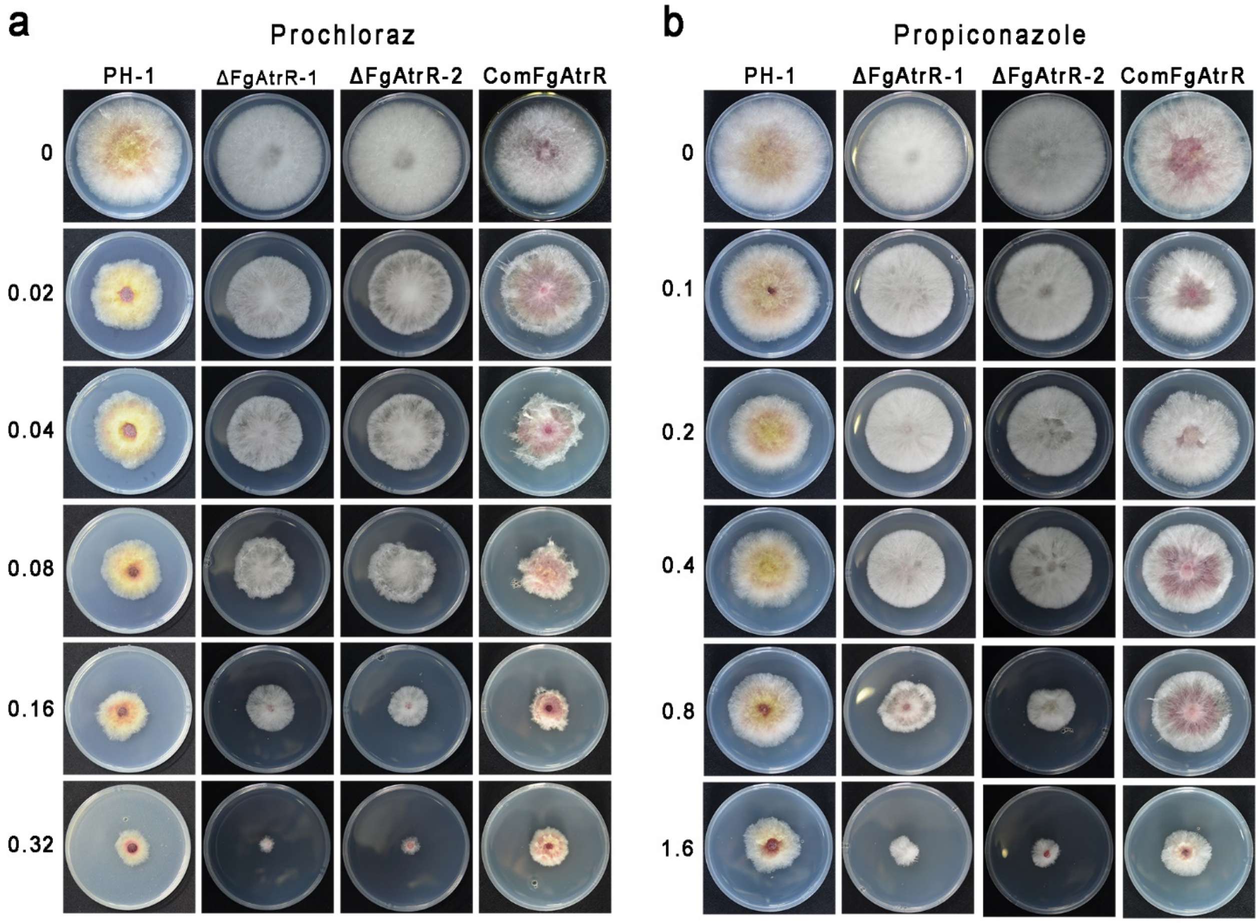

Figure S6 The growth inhibition effect of azole fungicide prochloraz (a) or propiconazole (b) on the *Fusarium graminearum* PH-1, FgAtrR deletion mutants, and FgAtrR-complemented strain at different concentrations. The concentration (mg/L) was labeled on the left side of each pane.
