# Supplementary material for: The Transcription Factor FgAtrR Regulates Asexual and Sexual Development, Virulence, and DON Production and Contributes to Intrinsic Resistance to Azole Fungicides in Fusarium graminearum
Source: Biology (Basel). 2022 Feb 18;11(2):326. doi: 10.3390/biology11020326 (PMC8869466; doi:10.3390/biology11020326)
Supplement: Supplementary file 1 [file biology-11-00326-s001.zip › Supplementary Materials/Table S1 Primers used in this study.docx]

Table S1 Primers used in this study

| **Primer name** | **Sequence (5′-3′)** | **Application** |
| --- | --- | --- |
| FgAtrR-AF | TAGACACGCTACGCCACG | To amplify the upstream fragment of *FgAtrR* |
| FgAtrR-AR | ttgacctccactagctccagccaagccATGCCTGGGACCTGGAAA |  |
| FgAtrR-BF | gaatagagtagatgccgaccgcgggttATGCCCATCGTGCCTAAC | To amplify the downstream fragment of *FgAtrR* |
| FgAtrR-BR | GCTACCTATCTCGGTTTCTTC |  |
| HYG-F | GGCTTGGCTGGAGCTAGTGGAGGTCAA | To amplify the 5' terminal fragment (HY) of the hygromycin resistance gene *HPH* |
| HY-R | GTATTGACCGATTCCTTGCGGTCCGAA |  |
| YG-F | GATGTAGGAGGGCGTGGATATGTCCT | To amplify the 3' terminal fragment (YG) of the hygromycin resistance gene *HPH* |
| HYG-R | AACCCGCGGTCGGCATCTACTCTATTC |  |
| FgAtrR-K1-F | GAGGTAAACGGATGGTGG | For identification of FgAtrR deletion transformants |
| FgAtrR -K1-R | AATCTGAAATAAAGGGAGG |  |
| FgAtrR -K2-F | TGTAGGAGGGCGTGGATA | For identification of FgAtrR deletion transformants |
| FgAtrR -K2-R | CGCTTAGGTGTTGCTGGT |  |
| FgAtrR -M-F | TCGGCTATGATGCTTGAA | For identification of FgAtrR deletion transformants |
| FgAtrR -M-R | ATTGTCGTGAGGCAGTTAT |  |
| FgAtrR -south-F | CTCTGGAGGAATCGTAGCA | To amplify the DNA fragment used as the probe for Southern blot analysis |
| FgAtrR -south-R | CCGACCAAAGCGTAATGT |  |
| ComAtrR-F | actcactatagggcgaattgggtactcaaattggttACGAGGTAAACGGATGGTGG | To amplify the DNA fragment used for construction of FgAtrR-complemented strain |
| ComAtrR-R | caccaccccggtgaacagctcctcgcccttgctcacGGAGGTAGGTTAGGCACGATG |  |
| ComAtrR-GFP-F | cagatcttggctttcgtaggaacccaatcttcaaaATGGACCACATGGCTTTCCAGG | To amplify the DNA fragment with used for construction of FgAtrR-complemented strain |
| ComAtrR-GFP-R | accccggtgaacagctcctcgcccttgctcacATCACCACCAAAGTTACCATTTTCAAC |  |
| Actin-RT-F | TAGCATCGCATCTCATCAC | For qPCR analysis of actin gene in *F. graminearum* |
| Actin-RT-R | CAGCAACTTCTTCCTCCAT |  |
| AurF-RT-F | TAACACTGCTGCTGACAT | For qPCR analysis of *aurF* gene in *F. graminearum* |
| AurF-RT-R | CACATACTTGCGGTAGGT |  |
| AurJ-RT-F | GCCAGTTATCACACCATCTT | For qPCR analysis of *aurJ* gene in *F. graminearum* |
| AurJ-RT-R | GTTGCTTGTCATTGCCATAC |  |
| PKS12-RT-F | CTGCTGTGTTAGTGATATGC | For qPCR analysis of *PKS12* gene in *F. graminearum* |
| PKS12-RT-R | GAGACGCTGGTTGCTATA |  |
| CYP51A-RT-F | CCGACATTACCGAAGAAC | For qPCR analysis of *FgCYP51A* gene in *F. graminearum* |
| CYP51A-RT-R | ATTGAGTGGATGGAAGAGT |  |
| CYP51B-RT-F | TCCGTCGTCCTCAATGTC | For qPCR analysis of *FgCYP51B* gene in *F. graminearum* |
| CYP51B-RT-R | CGTATGTGATGGTGCTTCC |  |
| CYP51C-RT-F | CTGAGTTAGAGCACGATGGAAT | For qPCR analysis of *FgCYP51C* gene in *F. graminearum* |
| CYP51C-RT-R | GACGATGGACAAGATGATGATGA |  |
| Tir5-RT-F | GATGGAGAACTGGATGGT | For qPCR analysis of *TRI5* gene in *F. graminearum* |
| Tri5-RT-R | TGCTTAGACGAGTGTAGG |  |
| Tri6-RT-F | CTGTCGCTACTCAGAATG | For qPCR analysis of *TRI6* gene in *F. graminearum* |
| Tri6-RT-R | TTGTTGTCCTTCCTTGTC |  |
| Tri11-RT-F | CTGTCGCTACTCAGAATG | For qPCR analysis of *TRI11* gene in *F. graminearum* |
| Tri11-RT-R | TTGTTGTCCTTCCTTGTC |  |
